# Managing Recurrent Rectal Variceal Bleeding Secondary to Portal Hypertension With Liquid Embolics

**DOI:** 10.7759/cureus.21614

**Published:** 2022-01-25

**Authors:** Akram Al-Warqi, Rahil H Kassamali, Mohammed Khader, Ayman Elmagdoub, Ali Barah

**Affiliations:** 1 Radiology, Hamad Medical Corporation, Doha, QAT; 2 Interventional Radiology, Hamad Medical Corporation, Doha, QAT; 3 Aston Medical School, Aston University, Birmingham, GBR

**Keywords:** amplatzer plug device, trans-splenic, portal vein thrombosis, bato, liver cirrhosis, portal hypertension, sclerotherapy, glue, liquid embolic, rectal varices

## Abstract

Rectal variceal bleeding is one of the rarer manifestations of portal hypertension caused by chronic liver disease. The management of these varices is very challenging. Our patient had portal vein thrombosis and presented with chronic recurrent rectal bleeding requiring transfusion secondary to rectal varices. The patient was treated from trans-splenic access with liquid embolics (sclerotherapy and glue) without balloon occlusion, leading to the successful cessation of his bleeding. Access hemostasis was achieved using a vascular plug in the access tract. There are no clear guidelines for the management of these patients. If rectal varices cannot be managed by colonoscopy, this approach to embolization with liquid embolic is an excellent minimally invasive alternative.

## Introduction

Portal hypertension secondary to liver cirrhosis commonly leads to portosystemic shunts and varices [1]. These shunts can be from the inferior mesenteric vein to the middle and inferior rectal veins leading to rectal varices that can cause chronic recurrent bleeding or acute life-threatening bleeding [2]. Rectal varices can be differentiated from hemorrhoids as they usually originate more than 4 cm above the anal verge and have a more tubular appearance below the colonic mucosa rather than the lumpy appearance of hemorrhoids. In addition, cross-sectional imaging often demonstrates the imaging features of liver disease and secondary signs of portal hypertension [3,4]. The prevalence of rectal varices is 38%-56% in patients with cirrhosis or 63%-94% in a patient with extrahepatic portal vein obstruction [5]. Although the prevalence of rectal varices is relatively high in this subset population, it is rare that it can cause significant bleeding in around 0.5%-5% of patients [6]. Bleeding rectal varices are most commonly diagnosed and treated with colonoscopy [7]. If this fails, further imaging with CT or MRI is required to identify the inflow and outflow of the varices [8,9]. Although there are multiple options, there is no clear guideline on how to reduce the risk of catastrophic bleeding in these patients.

## Case presentation

**Figure 1:**
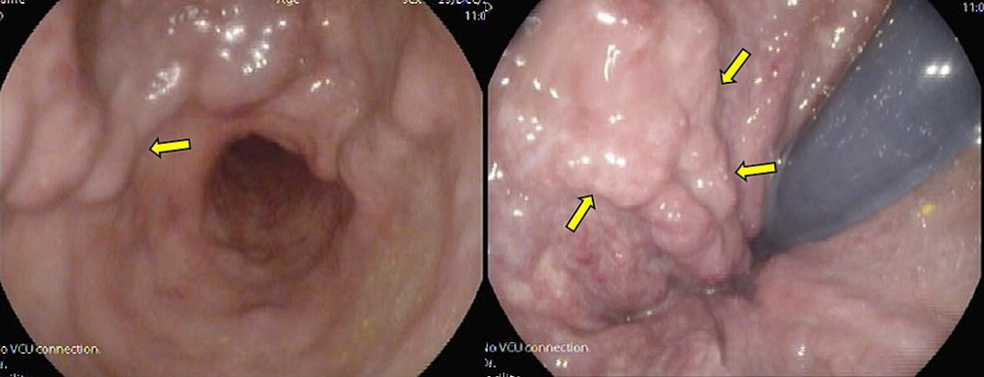
Selected colonoscopy images demonstrating submucosal rectal varices (yellow arrows).

**Figure 2:**
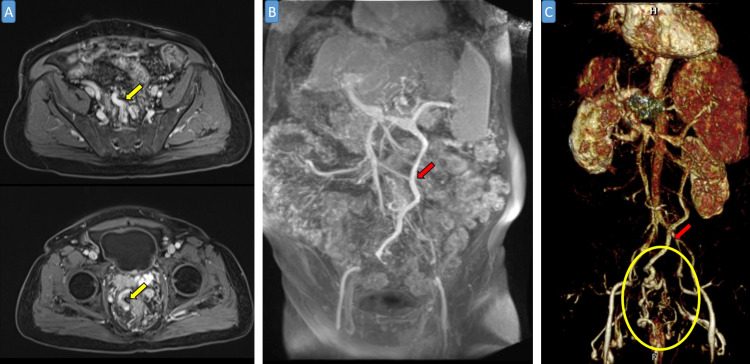
Abdominal MR venography: axial T1-weighted imaging post-contrast (A), coronal abdominal MIP reformat (B), and coronal 3D reformat (C). Imaging shows significant dilatation of perirectal varices (yellow arrows) as well as dilated tortuous inferior mesenteric vein (red arrows in B and C). The tortuous dilated inferior mesenteric vein in continuous with the rectal varices (yellow circle in C).

A 67-year-old male with a history of nonalcoholic steatohepatitis (NASH) cirrhosis and stigmata of portal hypertension presented with intermittent, recurrent lower GI bleeding. He presented to the emergency department with hemoglobin (Hb) of 7 mg/dL from a baseline of 10 mg/dL. The patient was resuscitated and transfused two units of blood. The bleeding spontaneously stopped. He had several previous episodes of significant rectal bleeding, which were self-limiting. This patient had multiple comorbidities, including hepatocellular carcinoma (HCC) and partial portal vein thrombosis. The portal vein thrombus did not show enhancement on MRI and demonstrated no uptake on positron emission tomography (PET), implying that it was a nontumor thrombus. He had been taking anticoagulation and antiplatelet agents to treat the thrombosis; however, this was stopped on admission. A colonoscopy was carried out 48 hours after admission. These demonstrated extensive rectal varices, but no focal bleeding point. Due to this, no endoscopic intervention was performed (Figure 1). MR venography was carried out, which demonstrated partial portal vein thrombosis with collateralization, a dilated inferior mesenteric vein (IMV) with a shunt to the rectal veins, and a mature network of large tortuous draining varices (Figure 2). As the patient was not a candidate for correction of the portal pressure with transjugular intrahepatic portosystemic shunt (TIPS) due to portal vein thrombosis and multiple comorbidities, a decision was made to embolize the inflow to the varices.

**Figure 3:**
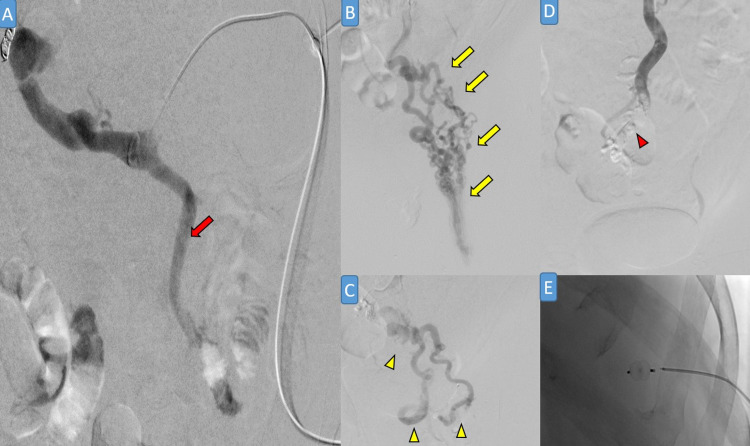
Portogram through splenic vein access showing dilated tortuous inferior mesenteric vein (red arrow in A) supplying severely dilated tortuous perirectal/perianal veins (yellow arrows in B). After administering sodium tetradecyl sulfate (STS) foam, there are persistent tortuous rectal varices (yellow arrowheads in C). N-Butyl cyanoacrylate (glue) was added, leading to total varix occlusion (red arrowhead in D). A splenic Amplatzer vascular plug was used to occlude splenic access to prevent bleeding (E).

**Figure 4:**
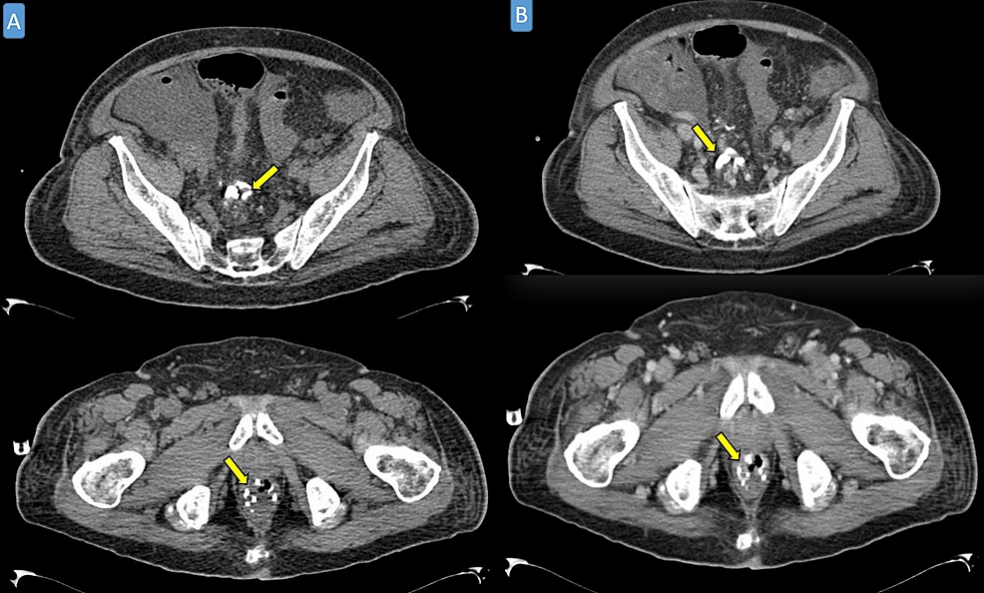
Post-procedure axial abdominal CT scan in plain (A) and post-contrast (B) displaying perirectal/perianal multiple hyperdensities of embolization materials (yellow arrows).

The procedure was carried out under conscious sedation. As the portal vein was occluded, a puncture of the intrasplenic splenic vein was performed with a 20-gauge (G) needle. Access was secured with a 5 French (Fr), 11-cm sheath (Cordis, CA, USA). A portogram was performed with a 4 Fr pigtail catheter (Boston Scientific Corporation, MA, USA) via an injector pump (30 mL at 10 mL/second) (Figure 3A). This demonstrated the varices filling via the IMV with drainage via the iliac veins bilaterally (Figure 3B). A 4 Fr vertebral catheter (Terumo, Tokyo, Japan) and hydrophilic angle tip guidewire (Terumo, Tokyo, Japan) were used to catheterize the IMV. As the flow was going toward the systemic venous circulation with no reflux toward the portal system, no balloon occlusion was required. Embolization was initially performed with 8 mL of 3% sodium tetradecyl sulfate (STS) mixed with 5 mL air and 2 mL lipiodol to form a foam. Although this reduced the number and size of the varices, complete occlusion was not achieved, and it was difficult to predict if sclerosant was moving across the shunt into the venous circulation (Figure 3C). Further embolization was therefore performed with N-butyl cyanoacrylate mixed with lipiodol in a 1:3 ratio (total volume: 6 mL). Post-embolization venogram demonstrated complete occlusion of the varices (Figure 3D). Due to the vascular nature of the spleen, the tract was embolized with a 6 × 11 mm Amplatzer plug type 4 (Abbott, IL, USA) (Figure 3E). No immediate complications were encountered. Twenty-four hours post-procedure, a CT scan was performed. This demonstrated high attenuation lipiodol embolic material within the rectal varices and distal IMV, with complete occlusion (Figure 4). No high attenuation embolic material had passed into the draining iliac veins. Over the next seven days, the patient had no further episodes of rectal bleeding, and his hemoglobin remained stable. His anticoagulation was recommenced, and further treatment of his HCC was also planned. He was discharged back to outpatient follow-up.

## Discussion

The most crucial part of the management of rectal varices is resuscitation and coagulopathy correction. This could be provided by crystalloids and packed red blood cell transfusion to maintain blood pressure between 90 and 100 mmHg, heart rate below 100 beats/minute, and hemoglobin level around 8 g/dL (hematocrit: 24%) [10,11]. There are no randomized controlled trials to support the use of vasoactive drugs such as vasopressin, octreotide, or terlipressin [10].

The endoscopic options for the management of rectal varices include injection sclerotherapy and endoscopic band ligation. Endoscopic ultrasound can be used to inject cyanoacrylate glue [2]. If endoscopic management fails or is technically not possible, the patient is referred to interventional radiology [2]. There are limited open surgical options for these patients [10].

If a patient is clinically suitable for transjugular intrahepatic portosystemic shunt (TIPS), this is an excellent option to treat the underlying portal hypertension. TIPS can sometimes be combined with venous obliteration procedures to further reduce the risk of repeat bleeding. However, TIPS is not suitable for every patient [12]. The interventional radiologist has some other techniques available to them to manage varices related to portal hypertension in patients who are not a candidate for TIPS. The procedures can be carried out from a transvenous approach or portal venous approach. These conventionally include balloon-occluded retrograde transvenous obliteration (BRTO) and balloon-occluded antegrade venous obliteration (BATO), which have been described for the management of gastric varices [13].

For this case, we used a modification of the BATO procedure. The approach was trans-splenic into the portal venous circulation; however, no balloon occlusion was required from the portal side as the flow of blood was definitively toward the systemic venous circulation with no reflux toward the portal vein. The initial choice to use 3% sodium tetradecyl sulfate mixed with lipiodol and air was based on the conventional description of the BATO procedure [14]. This did not however provide sufficient occlusion, and it was difficult to visualize if sclerosant was crossing the shunt into the venous circulation. The choice of embolic was changed to N-butyl cyanoacrylate mixed with lipiodol as this has a higher viscosity, giving more predictable, slow forward flow and radiopaque casting within the varices. Onyx 18 Liquid Embolic System (Medtronic, MN, USA) could also have been used in this scenario; however, this embolic agent is currently not available in our center [15].

This case highlights a modification to a previously well-described procedure. The learning points can be taken from an alternative access route if portal vein thrombosis is present, how splenic access can be plugged to achieve hemostasis, the choice of embolic agent, and that balloon occlusion is not always required.

Rectal varices secondary to portal hypertension causing chronic intermittent bleeding are a rare occurrence, making decision-making related to the treatment challenging. In addition, when a choice is made to provide an intervention, there are several options with no consensus on the best approach and best embolic material. In this case, the patient had medical management, endoscopic management, and eventually interventional radiology management. We chose to use liquid embolics without balloon occlusion, and of these, N-butyl cyanoacrylate combined with lipiodol was the most effective. We would recommend N-butyl cyanoacrylate as an embolic material for cases of rectal varices treated from a portal approach.

## Conclusions

The management of rectal varices can be challenging. In our case, modifying the previously described BATO procedure and using mixed liquid embolic agents helped resolve this patient's recurrent bleeding. Further evidence is still required in this area to help create more definitive guidelines.
